# Antiperiodontitis Effects of *Magnolia biondii* Extract on Ligature-Induced Periodontitis in Rats

**DOI:** 10.3390/nu11040934

**Published:** 2019-04-25

**Authors:** Hae Jin Lee, Dong-Ryung Lee, Bong-Keun Choi, Seung Hwan Yang

**Affiliations:** 1 Department of Biotechnology, Chonnam National University, Yeosu 59626, Korea; hjlee@nutrapharm.co.kr; 2 Nutrapharm Tech, Jungwon-gu, Seongnam, Gyunggi 13201, Korea; drlee@nutrapharm.co.kr (D.-R.L.); cbcbcbk@nutrapharm.co.kr (B.-K.C.)

**Keywords:** antiperiodontitis, inflammatory cytokine, alveolar bone resorption, *Magnolia biondii*, periodontal tissue

## Abstract

Over the past decades, periodontitis has become a rising health problem and caused various diseases. In the many studies shows that some extracts and compound to the prevention and treatment of periodontitis. This study focuses on the effects of inhibition of gingival damage and alveolar bone loss. The aim of this study was to evaluate the protective effects of *Magnolia biondii* extract (MBE) against ligature-induced periodontitis in rats. A ligature was placed around the molar teeth for 8 weeks, and MBE was administered for 8 weeks. Gingival tissue damage and alveolar bone loss were measured by microcomputed tomography (CT) analysis and histopathological examination. Serum Interluekin-1 β (IL-1β), tumor necrosis factor-α (TNF-α), cyclooxygenases-2 (COX-2), and receptor activator of nuclear factor–κB ligand (RANKL) levels were investigated using commercial kits to confirm the antiperiodontitis effects of MBE. We confirmed that ligature-induced periodontitis resulted in gingival tissue damage and alveolar bone loss. However, treatment for 8 weeks with MBE protected from periodontal tissue damage and downregulated serum inflammatory cytokine factors and RANKL levels. These results suggest that MBE exerts antiperiodontitis effects by inhibiting gingival tissue destruction and alveolar bone loss through regulation of anti-inflammatory cytokines in periodontitis-induced rats.

## 1. Introduction

Periodontitis is a chronic inflammatory disease that gradually destroys the periodontium including the gums, cementum periodontal ligament, and alveolar bone surrounding and supporting the teeth [1,2]. It was reported that periodontal disease affects 20–50% of the global population, and 47.2% of the US population over 30 years of age suffers from a certain degree of periodontitis [2,3]. The high prevalence rates of periodontal diseases have made it a serious worldwide health problem today. Periodontal disease also increases the risk of type II diabetes, hypertension, cardiovascular disease, and metabolic syndrome [3,4]. Moreover, people have been exposed to the risk of oral squamous cell cancer recently due to low fruit and vegetable intake, lack of vitamin intake, and abuse of alcohol and tobacco, and new cases are reported every year [5]. Also, chronic inflammation causes odontoma, which is considered as the most common odontogenic tumor of the oral cavity. It affects tooth tissue, such as enamel, dentin, pulp, and cementum [6]. Prevention and proper management of periodontal disease is therefore of importance. Corticosteroids and NSAIDS have been used to treat periodontal diseases. However, it has been reported some adverse effects such as gastrointestinal bleeding, a reduction in platelet function and when long-term use, the immune defense system decreased. Therefore, it is needed for safe and effective materials without side effects [7].

Previous studies demonstrated that the main cause of periodontitis is bacterial plaque accumulation [1]. Plaque accumulation causes an inflammatory process and activates the host immune response in periodontal tissue by secreting proinflammatory cytokines such as tumor necrosis factor-alpha (TNF-α), and Interluekin-1 β (IL-1β). These cytokines then stimulate the production of secondary-mediators, including chemokines such as cyclooxygenases-2 (COX-2) [8]. These factors have a central role in the destruction of periodontal tissue by causing periodontal pocket formation, connective tissue damage, and alveolar bone resorption [9]. Alveolar bone loss is a typical feature of periodontitis, which is dependent upon the balance between osteoclast-mediated bone resorption and osteoblast-mediated bone formation [10]. It has been demonstrated that bone resorption is induced by osteoclasts and is stimulated by the receptor activator of nuclear factor–Β ligand (RANKL) [11].

*Magnolia biondii* (MB) is one of a plant species belonging to *Magnolia flos* (MF) and has traditionally been used to treat nasal congestion with headache, sinusitis, and allergic rhinitis [12,13]. It is listed in the Chinese and Korea Pharmacopoeia and is known to have a wide range of pharmacological properties, including anti-inflammatory, antiallergic, antiproliferative, antifungal, and antimicrobial [13,14]. Therefore, we hypothesized that the anti-inflammatory activity of MB extract (MBE) would inhibit gingival destruction and inhibit osteoclast differentiation would reduce alveolar bone resorption. To test of this hypothesis, we examined the antiperiodontitis effects of MBE by investigating its anti-inflammatory activities on ligature-induced periodontitis in rats.

## 2. Materials and Methods

### 2.1. Sample Preparation

The MB extract (MBE) was provided from Nutrapharmtec (Seongnam, South Korea). The dried MB was extracted with aqueous ethyl alcohol and filtered. The extracts were concentrated in a vacuum evaporator, and the concentrate was sterilized and cooled. The residue was dried, and a powder was obtained. MBE was dissolved in saline and subsequently used for in vivo study.

### 2.2. Animals

Twenty-five male 6-week-old Crl;CD(SD) rats were purchased from Orient-bio Co. (Seongnam, South Korea). After acclimation for 7 days, healthy animals were selected and used for experimentation. The animals were given free access to food and water. This study was conducted under the conditions of a 12-h light-dark cycle at 23 ± 3 °C (8:00 AM to 8:00 PM), 55 ± 15% humidity, and illumination at 150 to 300 lux. The animal experiments were carried out in accordance with the national guidelines for the care and use of laboratory animals approved by the animal Ethics committee (permission number: KNOTUS-IACUC-17-KE-333) of KNOTUS Inc (Guri, Korea). We monitored changes in body weight once a week and observed changes in feed and water intake. To improve animal well-being, we provided a sanitary environment to prevent disease and proper breeding and management.

### 2.3. Ligature-Induced Periodontal Disease and Drug Administration

Animals were anesthetized with Zoletil 50 (Virbac, France) and xylazine (Rompun^®^, Germany) by intraperitoneal injection. After anesthesia, the rats’ mouths were kept open to facilitate access to the posterior teeth of the mandible. A 4-0 silk ligature was placed around the right second molar of the mandible for 8 weeks to induce periodontitis. After induction of periodontitis, the rats were divided into six groups: (1) nonligature control + vehicle, (2) ligature control + vehicle, (3) ligature + doxycycline 20 mg/kg, (4) ligature + MBE 100 mg/kg, and (5) ligature + 400 mg/kg. The drugs were dissolved in distilled water and orally administered once a day for 8 weeks. The total volume of daily gavage was 10 mL.

### 2.4. Microcomputed Tomography (Micro-CT) Analysis

All rats were anesthetized, and their mandibular jaws were scanned using a micro-CT (SCANCO Medical, Switzerland) at 8 weeks and 16 weeks. The cement–enamel junction (CEJ)–alveolar bone crest (ABC) distance and furcation involvement in the periodontitis-induced area in the images were measured to confirm alveolar bone loss and tissue damage. Distances were analyzed using the built-in instrument software. The CEJ–ABC distance was expressed as the mean value of the distance between the left and right CEJ to the ABC of the mandibular second molar regions. The furcation involvement was also analyzed by sliced micro-CT images taken using the software in the second molar regions. The device was set to 70 kv with 114 A energy and an integration time 200 ms per projection. It yielded a series of ~420 consecutive 25 m slices that create serial incisor teeth to the mandible. The images were produced with a voxel size of 25 m.

### 2.5. Measurement of Gingival Index and Tooth Mobility

Animals were checked for ligation status, gingival bleeding, and the degree of erosion per week after periodontal disease, according to the following criteria; Score 0, normal gingiva; Score 1, mild inflammation, slight edema, minor change in color, and absence of bleeding on probing; Score 2, moderate inflammation, edema, glazing, redness, and bleeding on probing; and Score 3, severe inflammation, extreme redness, presence of ulcers, edema, and severe bleeding. The Gingival Index was used to assess the degree of inflammation in the gingiva. Tooth mobility was scored according to the following scale; Score 0, no mobility; Score 1, slight mobility (vestibular–palatal); Score 2, severe mobility (vestibular–palatal and mesial-distal); and Score 3, severe mobility (vertical, the tooth moves in and out of the socket).

### 2.6. Histological Analysis and Inflammation Score of Periodontal Tissues

The rats were sacrificed after the end of experimentation at 16 weeks and immediately dissected in their mandibular molars, alveolar bone (AB), and surrounding soft tissues. The tissues were fixed in 4% formaldehyde (pH 7.5) overnight at 4 °C and then transferred to a decalcifying solution with 0.5 M EDTA-Na (pH 7.5–8.0) for 4 weeks. The tissues were embedded in paraffin, and serial mesiodistal sections (5 μm) were stained with hematoxylin-eosin. Histopathological changes in stained tissues were observed using an optical microscope (Olympus BX53, Japan). The inflammation scoring system used for the determination of periodontal status.

### 2.7. Serum Analysis

After 16 weeks of treatment, rats were anesthetized, and blood was collected. Blood samples were centrifuged at 2000 × g for 15 min at 4 °C for serum collection. The separated serum was stored at −80 °C until analysis. Serum levels of IL-1β, TNF-α (Invitrogen, USA), COX-2 (CUSABIO, USA), and RANKL (LSBio, USA) were determined using commercial kits according to the manufacturer’s instructions.

### 2.8. Statistical Analysis

Data were expressed as mean ± standard error and were analyzed with the SPSS Statistics 22.0 (SPSS Inc., Chicago, IL, USA) software. The different treatment groups were compared using Student’s *t-*test and one-way analysis of variance followed by multiple comparisons with Dunnett’s post hoc test using Origin 7.0 software (Microcal, MA, USA). Differences were considered statistically significant at *p* < 0.05 and *p* < 0.01.

## 3. Results

### 3.1. Micro-CT Analysis

**Figure 1:**
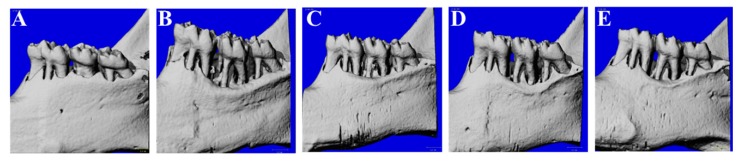
Effects of *Magnolia biondii* extract (MBE) on alveolar bone loss and tissue damage. The images of bone surrounding three molars were analyzed by three-dimensional microcomputed tomography and are shown here representative of each group: (**A**) non ligature-control, (**B**) ligature-control, (**C**) doxycycline 20 mg/kg, (**D**) MBE 100 mg/kg, and (**E**) MBE 400 mg/kg. The scale bar = 1.0 mm.

The periodontitis-induced rats were administered drugs for eight weeks, and the CEJ–ABC distance and furcation involvement before (week 8) and after (week 16) treatments were compared using micro-CT images. Representative images of all sample periodontal tissue results are shown in Figure 1. As shown in Table 1, the CEJ–ABC distance and furcation involvement in the ligature control group were significantly higher at eight weeks, at 0.098 mm and 0.041 mm, respectively. In the nonligature group, the CEJ–ABC distance had decreased by 0.007 mm, and the Furcation involvement had increased by 0.009 mm. We confirmed that the CEJ–ABC distance and furcation involvement levels in the ligature control group gradually increased over eight weeks and that these was significantly higher than in the nonligature group. However, levels were dramatically lowered upon doxycycline administration, at 0.066 mm and 0.030 mm, respectively, decrease in CEJ–ABC distance of 0.039 mm and a decrease in furcation involvement of 0.024 mm. In addition, MBE 100 mg/kg and 400 mg/kg treatment reduced the CEJ–ABC distance and furcation involvement in a dose-dependent manner. Administration of 400 mg/kg MBE effectively lowered the CEJ–ABC distance, similar to the effects seen with doxycycline administration.

### 3.2. Effects of MBE on Gingival Index (GI) and Tooth Mobility (TM) Measurement in Periodontitis Rats

**Figure 2:**
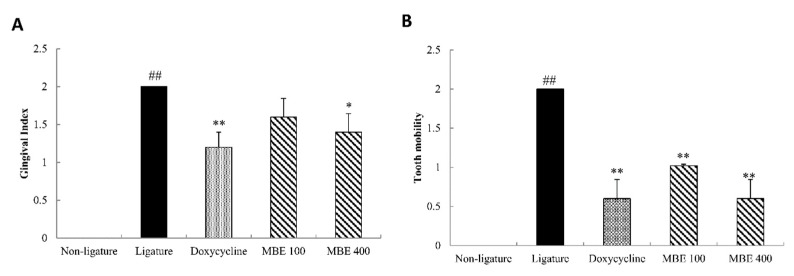
Effects of MBE on Gingival Index and Tooth mobility. The gingival index (**A**) and tooth mobility (**B**) were examined once a week after induced periodontal diseases. The data were evaluated at 16 weeks and are presented as mean ± SEM. *p < 0.05, **p < 0.01, compared with ligature control group; ^##^*p* < 0.01, compared with nonligature control group (*n* = 5/group).

The gingival index and tooth mobility were measured in rats, and the results are showed in Figure 2. The mean Gingival Index in the ligature control group was measured as 2, which was significantly higher than in the nonligature control. Doxycycline treatment reduced the Gingival Index to 1.2. Treatment with 100 mg/kg MBE also decreased the Gingival Index and treatment with 400 mg/kg MBE statistically lowered the gingival index to 1.4. In the ligature control group, tooth mobility was measured as 2, which was significantly higher than in the nonligature group. Doxycycline treatment dramatically reduced tooth mobility to 0.6 compared to the ligature control group. MBE treatment exhibited a statistically dose-dependent decrease: the results of 400 mg/kg MBE were similar to those of doxycycline administration.

### 3.3. Effects of MBE on Histological Analysis and Inflammation Score

**Figure 3:**
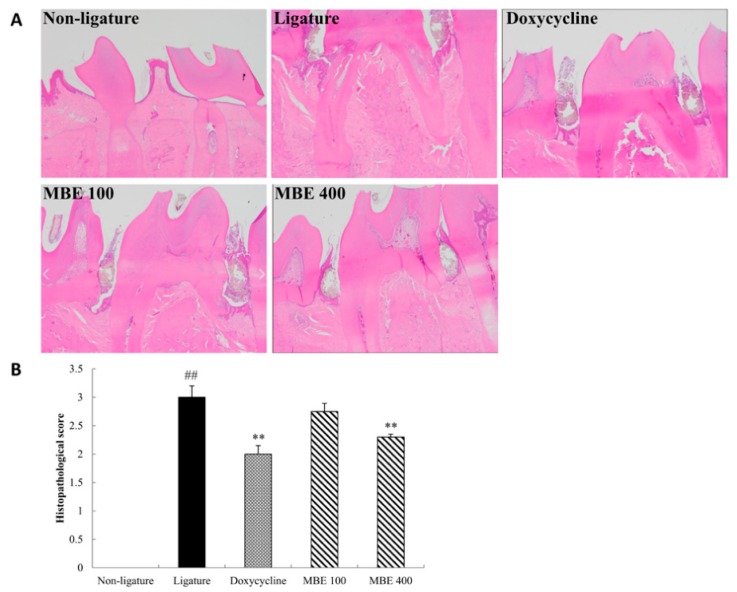
Effects of MBE on histological observation of inflammation and bone loss status in periodontitis rats. (**A**) Mandible tissues including molars were stained with hematoxylin-eosin and (**B**) scored according to periodontal status. The stained sections were examined at a magnification of 100 x using light microscopy (Olympus BX53, Japan). The data are presented as mean ± SEM. **p < 0.01, compared with ligature control group; ^##^*p* < 0.01, compared with nonligature control group (*n* = 5/group).

After the end experimentation, periodontal tissues were analyzed according to the inflammation score found in Table 2. The ligature control rats exhibited gingival epithelium erosion and moderate inflammatory cell infiltration. In contrast, periodontal tissues in the nonligature control group showed no lesions. Pathologic analysis of damaged periodontal tissues demonstrated that the administration of doxycycline significantly improved the degree of hyperplasia, inflammation, and periodontal ligament damage in the periodontal epithelium as compared with the ligature control group (Figure 3). Treatment with MBE 100 mg/kg decreased alveolar bone damage and inflammation erosion and showed improved periodontal conditions than the ligation control group. Treatment with 400 mg/kg diminished ligature-induced bone loss, histological changes, and inflammatory cell infiltration as compared with the ligature control group.

### 3.4. Effects of MBE on Serum Analysis in Periodontitis Rats

**Figure 4:**
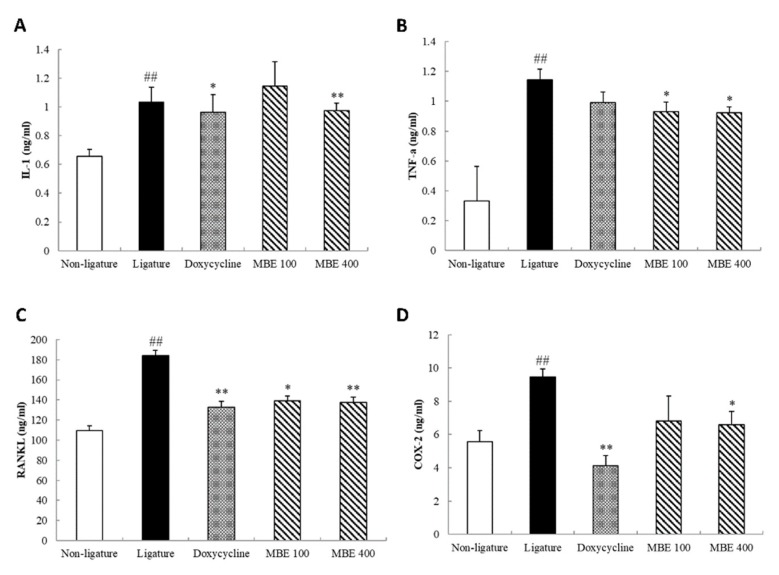
Effects of MBE on serum biochemistry in rats with ligature-induced periodontitis. The collected serum was analyzed for levels of (**A**) IL-1β, (**B**) TNF-α, (**C**) COX-2, and (**D**) RANKL using commercial ELISA kits. The data are presented as mean ± SEM. *p < 0.05, **p < 0.01, compared with ligature control group; ^##^*p* < 0.01, compared with nonligature control group (*n* = 5/group).

The results of periodontitis rat serum analysis are presented in Figure 4. Serum levels of IL-1β, TNF-α, COX-2, and RANKL were significantly lower in the nonligature rats than in the ligature control rats. Serum IL-1β levels in the doxycycline treatment group were reduced, and rats treated with 400 mg/kg MBE also showed significantly decreased IL-1β levels compared with the ligature control group. Rats treated with 100 and 400 mg/kg MBE showed decreased TNF-α levels when compared with the ligature control groups. Increased serum levels of COX-2 induced by ligation were significantly reduced upon doxycycline administration, and MBE treatment with 100 and 400 mg/kg doses showed a dose-dependent decrease. The concentration of RANKL in the ligature in the groups treated with 100 and 400 mg/kg MBE showed a statistically significant reduction compared with the ligature control group. In addition, doxycycline administration significantly reduced the increased RANKL levels. 

## 4. Discussion

The present study is the first report to evaluate the antiperiodontitis effects in ligature-induced periodontitis in rats. The ligature for eight weeks resulted in severe damage of gingival tissue and alveolar bone loss. In this study, we treated the periodontitis rats with MBE and confirmed the recovery effects by inhibiting serum cytokines and RANKL.

Numerous studies have used the rat ligature-induced periodontitis model to investigate preventive measures for periodontitis. Rat molars have an anatomically similar structure to human teeth, and periodontitis by ligation can imitate human periodontal disease progression [15]. Periodontitis typically exhibits symptoms of gingival tissue inflammation and alveolar bone loss, and it is quite similar to human periodontitis [1,16]. Therefore, we administered MBE to periodontitis rats in this study and confirmed the antiperiodontitis of MBE effects by regulating the various inflammatory factors involved.

Ligature placement around the teeth imitates accumulation of plaque and leads to ulceration of the sulcular epithelium, facilitating connective tissue damage [17]. To investigate gingival tissue destruction and alveolar bone loss, we measured the CEJ–ABC distance and furcation involvements using Micro-CT analysis. CEJ–ABC distance is used as parameter for measurement of periodontal breakdown [18]. Furcation involvement is known to be affected by the presence of periodontal diseases: a large value indicates more extensive alveolar bone loss [19]. In this study, we confirmed that ligation surrounding the teeth causes significant gingival tissue damage and alveolar bone loss, and it increase the CEJ–ABC distance and furcation involvement. Additionally, the GI and TM were measured to assess gingival tissue destruction and alveolar bone loss. The GI index was significantly increased by inflammation and edema in the ligature control group and tooth mobility was also higher due to gingival tissue damage, including alveolar bone loss. However, MBE treatment diminished the gingival index and significantly lowered tooth mobility compared with the ligature control group. The findings of this study demonstrated that MBE administration directly inhibited the progression of periodontitis, by reducing gingival tissue destruction and alveolar bone loss.

Furthermore, to investigate the antiperiodontitis effects of MBE, we performed histopathological examination and quantified our findings. The ligature control group showed erosion and ulceration of gingival epithelium and moderate inflammatory cell infiltration [19]. Moreover, it was confirmed that the periodontal ligament and the alveolar bone were destroyed along with periodontitis progression. In the present study, treatment with 400 mg/kg MBE showed mild hyperplasia of the gingival epithelium and inflammation without alveolar bone loss when compared with the ligature control group.

**Figure 5:**
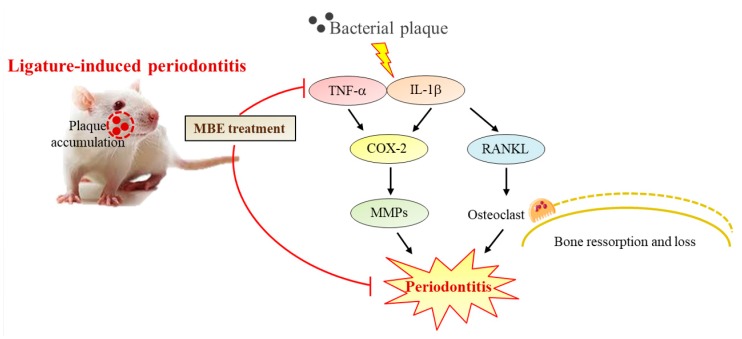
The proposed mechanism of *Magnolia biondii* extract’s antiperiodontitis effects.

The accumulation of plaques due to ligation causes gingival tissue inflammation, and the associated immune response is activated. The main cause of periodontitis, bacterial plaque, triggers production of key cytokines, such as IL-1β and TNF-α, from macrophages. IL-1β, which has a wide range of biological activities, is known to have a strong association with the Gingival Index and pocket depth, and levels of IL-1β were significantly lower in healthy gingival tissue than in inflamed tissue in periodontitis patients [20]. TNF-α causes tissue destruction and an erosive reaction in periodontitis, and increased TNF-α levels are known to promote cartilage collagen degradation and bone resorption [21]. COX-2 plays a role as a mediator in inflammatory pathways. The inflammatory response is strongly activated upon release of proinflammatory cytokines such as such as IL-1β and TNF-α [22,23]. These are also known to play an important role in the initiation and progression of periodontitis and can upregulate RANKL expression in periodontal cells and increase osteoclast formation [11,24]. These cytokines are produced by lymphocytes and stromal/osteoblastic cells, and are known to exhibit high activity in humans with periodontal disease. In addition, RANKL is essential for osteoclast precursor differentiation and plays an important role in periodontal bone resorption [11,25]. In this study, ligation-induced rats showed increased serum levels of IL-1β, TNF-α, COX-2, and RANKL. By contrast, MBE treatment decreased these serum indicators as compared with the ligature control group. These results suggest that MBE alleviates the progression of periodontitis by reducing gingival tissue destruction and alveolar bone resorption factors and the relevant mechanism of action of MBE in this study is shown in Figure 5.

## 5. Conclusions

Periodontitis caused by ligation with *Porphyromonas gingivalis* is triggered by inflammatory cytokine pathway and it destroys gingival tissue. In addition, cytokines activate osteoclasts to promote alveolar bone resorption and the alveolar bone destruction causes tooth loss.

Our results indicate that MBE treatment decreased CEJ–ABC distance and furcation involvement and also reduced gingival index and tooth mobility with periodontitis induced rats. MBE administration also confirmed the reduction of the inflammatory level of periodontal tissue by histopathological examination. Furthermore, MBE significantly inhibited gingival destruction and alveolar bone resorption and these results were evidenced by decreased levels of serum inflammatory cytokines and RANKL levels. However, this study has limitations that it used in vivo model, and needed to further experiment on the mechanism study in periodontal tissue. We will confirm the efficacy by identifying related mechanism and conducting further study of the patients with periodontitis. Such confirmation would suggest that MBE will be developed as a health functional food and medicine for preventing or treating patients with periodontal disease.

## Figures and Tables

**Table 1:** Effects of MBE on cement–enamel junction (CEJ)–alveolar bone crest (CEJ–ABC) distance and furcation involvement with micro-CT images analysis in periodontitis rats.

Treatment	8 Weeks		16 Weeks		Week 16 – Week 8	
	CEJ–ABC Distance (mm)	Furcation Involvement (mm)	CEJ–ABC Distance (mm)	Furcation Involvement (mm)	CEJ–ABC Distance (mm)	Furcation Involvement (mm)
Nonligature control	0.758 ± 0.089 ^1^	0.166 ± 0.019	0.751 ± 0.099	0.175 ± 0.02	−0.007 ± 0.012	0.009 ± 0.003
Ligature control	1.638 ± 0.161 ^##^	0.335 ± 0.018	1.736 ± 0.157 ^##^	0.377 ± 0.014 ^##^	0.098 ± 0.011 ^##^	0.041 ± 0.005 ^##^
Doxycycline	1.483 ± 0.056	0.348 ± 0.026	1.417 ± 0.046 ^**^	0.318 ± 0.030 ^**^	−0.066 ± 0.008 ^**^	−0.030 ± 0.003 ^**^
MBE 100	1.495 ± 0.143	0.334 ± 0.025	1.456 ± 0.140 ^*^	0.318 ± 0.026 ^**^	−0.030 ± 0.010 ^**^	−0.014 ± 0.001 ^**^
MBE 400	1.507 ± 0.056	0.346 ± 0.041	1.428 ± 0.047 ^**^	0.316 ± 0.043 ^*^	−0.061 ± 0.015 ^**^	−0.020 ± 0.001 ^**^

^1^ The data are presented as mean ± SEM. *p < 0.05, **p < 0.01, compared with ligature control group; ^##^*p* < 0.01, compared with nonligature control group.

**Table 2:** Inflammation assessment criteria for periodontal status.

Score	Degree	Lesions
0	Absence	None
1	Slight	Slight hyperplasia of gingival epithelium and inflammatory cell infiltration. No substantial changes in PDL or ABC
2	Mild	Mild hyperplasia of gingival epithelium and inflammation. Slight disruption of the PDL
3	Moderate	Erosion and ulceration of gingival epithelium and moderate inflammatory cell infiltration
4	Severe	Erosion and ulceration of gingival epithelium and severe inflammation
